# System Biology Investigation Revealed Lipopolysaccharide and Alcohol-Induced Hepatocellular Carcinoma Resembled Hepatitis B Virus Immunobiology and Pathogenesis

**DOI:** 10.3390/ijms241311146

**Published:** 2023-07-06

**Authors:** Vishal S. Patil, Darasaguppe R. Harish, Ganesh H. Sampat, Subarna Roy, Sunil S. Jalalpure, Pukar Khanal, Swarup S. Gujarathi, Harsha V. Hegde

**Affiliations:** 1 ICMR-National Institute of Traditional Medicine, Nehru Nagar, Belagavi 590010, India; vishalpatil6377@gmail.com (V.S.P.); ganeshsampat72272@gmail.com (G.H.S.); bhavsargam7744896491@gmail.com (S.S.G.); harshah@icmr.gov.in (H.V.H.); 2 KLE College of Pharmacy, Belagavi, KLE Academy of Higher Education and Research, Belagavi 590010, India; jalalpuresunil@rediffmail.com (S.S.J.); pukarkhanal58@gmail.com (P.K.)

**Keywords:** alcohol, hepatitis B, hepatocellular carcinoma, lipopolysaccharide, network pharmacology, rodent model

## Abstract

Hepatitis B infection caused by the hepatitis B virus is a life-threatening cause of liver fibrosis, cirrhosis, and hepatocellular carcinoma. Researchers have produced multiple in vivo models for hepatitis B virus (HBV) and, currently, there are no specific laboratory animal models available to study HBV pathogenesis or immune response; nonetheless, their limitations prevent them from being used to study HBV pathogenesis, immune response, or therapeutic methods because HBV can only infect humans and chimpanzees. The current study is the first of its kind to identify a suitable chemically induced liver cirrhosis/HCC model that parallels HBV pathophysiology. Initially, data from the peer-reviewed literature and the GeneCards database were compiled to identify the genes that HBV and seven drugs (acetaminophen, isoniazid, alcohol, D-galactosamine, lipopolysaccharide, thioacetamide, and rifampicin) regulate. Functional enrichment analysis was performed in the STRING server. The network HBV/Chemical, genes, and pathways were constructed by Cytoscape 3.6.1. About 1546 genes were modulated by HBV, of which 25.2% and 17.6% of the genes were common for alcohol and lipopolysaccharide-induced hepatitis. In accordance with the enrichment analysis, HBV activates the signaling pathways for apoptosis, cell cycle, PI3K-Akt, TNF, JAK-STAT, MAPK, chemokines, NF-kappa B, and TGF-beta. In addition, alcohol and lipopolysaccharide significantly activated these pathways more than other chemicals, with higher gene counts and lower FDR scores. In conclusion, alcohol-induced hepatitis could be a suitable model to study chronic HBV infection and lipopolysaccharide-induced hepatitis for an acute inflammatory response to HBV.

## 1. Introduction

Hepatitis B virus (HBV) is a member of the family Hepadnaviridae, possessing a 3.2 kb short genome with largely double-stranded DNA [1]. The human sodium taurocholate co-transporting polypeptide (NTCP) receptor and the viral envelope protein (HBsAg) interact in a remarkably species-specific manner to allow HBV to enter human hepatocytes [2]. Several liver diseases, including cirrhosis, hepatocellular carcinoma, and liver fibrosis, can develop in those with chronic HBV infection [2]. Despite significant advancements in the diagnosis, prevention, and treatment of chronic hepatitis B (CHB), over 296 million individuals worldwide still have the HBV infection and account for an estimated 820,000 deaths, mostly by cirrhosis and hepatocellular carcinoma (HCC) [3]. Injections of interferon and oral nucleoside analogs are used to treat persistent HBV infection [4]. Currently, HIV and HBV polymerase reverse transcriptase inhibitors are licensed treatments for HBV, and only 30–40% of individuals with chronic HBV (CHB) react to interferon therapy [4]. The World Health Organization (WHO) recommends entecavir and tenofovir for the treatment of CHB [3].

**Figure 1:**
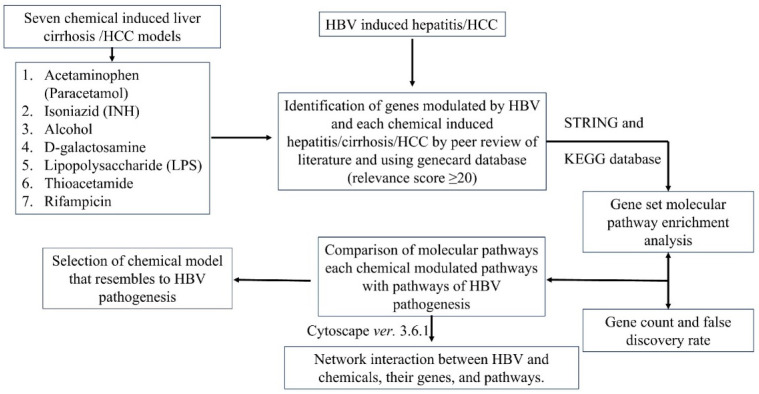
Workflow of the current study.

Several HBV cell culture-based systems, such as HepG2T14, HepG2.2.15, Q7 HBV-21, HepG2-4A5, and HepAD38, have been created and have been employed for cultivating the virus to conduct in vitro HBV inhibitor screening and investigate the control of viral replication [5]. In vivo models, however, have been and will continue to be essential for understanding the mechanisms underlying HBV pathogenesis, HBV-induced immune responses, and the testing of new antiviral therapeutic regimens [5]. Numerous in vivo models, such as those using chimpanzees, tupaiids, woodchucks, ducks, and woolly monkeys, have been produced since the “Australian antigen” was discovered. However, these animals are not routinely utilized as experimental hosts due to ethical and cost concerns [6]. Additionally, with the lack of small animal models that reproduce human-like HBV infections, it becomes extremely difficult to understand possible HBV disease mechanisms and develop efficient treatments [7]. Although an HBV mouse model may be suitable, this approach has several drawbacks. The 1.3-HBV transgenic mouse model, which has 1.3-HBV incorporated into the murine genome, is immune to HBV, does not cause liver damage, and does not produce cccDNA [8]. To maintain cells for six months, hydrodynamic injection (HDI)-based replication-competent HBV transgenic mice with HBV replicons, such as 1.2- or 1.3-HBV or HBV circle genomes, are hydrodynamically injected into mice. With the right vector, they can cause liver fibrosis and are expressed in 10–25% of murine hepatocytes post-inoculation. HBV genotype affects viral persistence [8]. Adeno-HBV transgenic mice were developed by injecting adenovirus vectors containing the HBV genome [9]. These mice become immunologically tolerant to HBV due to an altered T cell profile (an advantage for immunotolerant studies) and the absence of detectable cccDNA [10]. Apart from the above-mentioned models, various chemical-induced models (alcohol, acetaminophen, lipopolysaccharides, isoniazid, etc.) are also utilized to evaluate the hepatoprotective potential of compounds [11,12]. These chemically induced models alter multiple genes and pathways (PI3K-Akt, TNF, JAK-STAT, MAPK, Chemokine, NF-kappa B, TGF-beta signaling pathways, Apoptosis, and Cell cycle) and result in the development of hepatitis [13], which may be similar to that of HBV-induced hepatitis. Therefore, the goal of the current study was to combine gene set enrichment and network pharmacology analyses to pinpoint the chemical-induced hepatitis model that most closely resembles the pathophysiology of HBV; the current study’s workflow is presented (Figure 1).

## 2. Results

### 2.1. Identification of HBV-Associated and Chemically Induced Hepatitis Genes

Based on the literature review, 42 genes (from 36 articles) were obtained which were modulated in HBV-induced hepatitis. Similarly, from the GeneCards database, 1538 genes were obtained with a relevance score greater than 20. Among the 1538 genes, interferon-γ (IFN-γ) had the highest relevance score of 166.85, while KHK (Ketohexokinase) had the lowest relevance score of 20.00. Likewise, the genes obtained based upon the literature review were 66 genes for alcohol (from 65 articles), 38 genes for acetaminophen (from 11 articles), 30 genes for isoniazid (from 10 articles), 31 genes for D-galactosamine (from 14 articles), 44 genes for lipopolysaccharide (from 39 articles), 33 genes for rifampicin (from 26 articles), and 33 genes for thioacetamide (from 23 articles) were obtained and, similarly, were 407, 128,48, 52, 260, 24, 31 from the GeneCards database, respectively. From the list of genes for alcohol-induced hepatitis articles, alcohol dehydrogenase 1b (ADH1B), β-polypeptide had the highest relevance score of 98.78, and Serpin Family C member 1 (SERPINC1) had the lowest relevance score of 20.03. Interestingly TNF had the highest relevance scores of 75.06, 77.71, 69.39, 95.44, 51.13 for acetaminophen, isoniazid, D-galactosamine, lipopolysaccharide, and rifampicin-induced hepatitis, respectively, and for thioacetamide, IL6 had the highest relevance score of 55.21. UGT1A4, CYP2A6, CSF2, INRS, GSTM1, and BMP6 had the lowest relevance score of 20.08, 20.03, 20.47, 20.02, 20.0, and 20.02, respectively. The list of genes/protein molecules regulated by the HBV and each chemical-induced hepatitis which is obtained from a peer review of the literature (along with references [14,15,16,17,18,19,20,21,22,23,24,25,26,27,28,29,30,31,32,33,34,35,36,37,38,39,40,41,42,43,44,45,46,47,48,49,50,51,52,53,54,55,56,57,58,59,60,61,62,63,64,65,66,67,68,69,70,71,72,73,74,75,76,77,78,79,80,81,82,83,84,85,86,87,88,89,90,91,92,93,94,95,96,97,98,99,100,101,102,103,104,105,106,107,108,109,110,111,112,113,114,115,116,117,118,119,120,121,122,123,124,125,126,127,128,129,130,131,132,133,134,135,136,137,138,139,140,141,142,143,144,145,146,147,148,149,150,151,152,153,154,155,156,157,158,159,160,161,162,163,164,165,166,167,168,169,170,171,172,173,174,175,176,177,178,179,180,181,182,183,184,185,186,187,188,189,190,191,192,193,194,195,196,197,198,199,200,201,202,203,204,205,206,207,208,209,210,211,212,213,214,215,216,217,218,219,220,221,222,223,224,225,226,227,228,229,230,231,232,233]) and the GeneCards database are summarized in Supplementary Tables S1–S8.

### 2.2. Analysis of Genes Involved in Hepatic Toxicity

**Figure 2:**
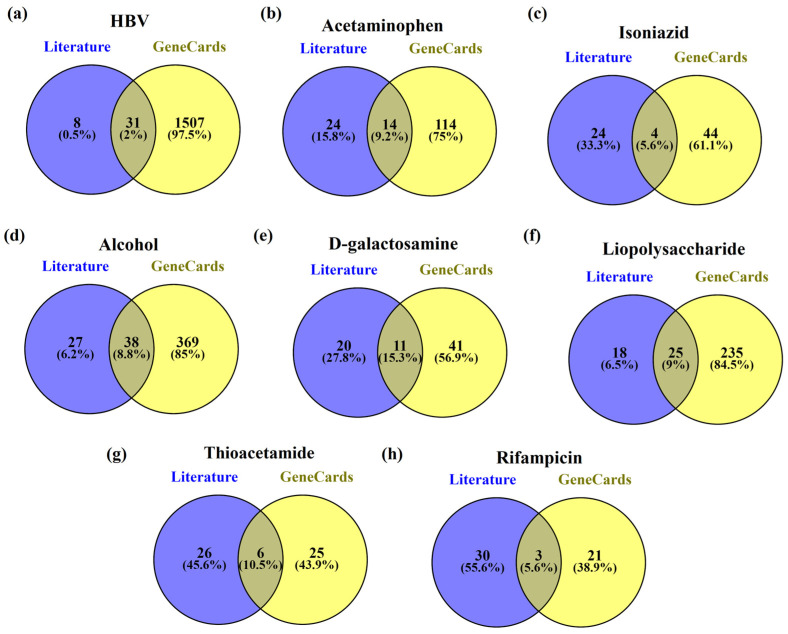
The genes common in both the literature review and GeneCards for (**a**) HBV, (**b**) acetaminophen, (**c**) isoniazid, (**d**) alcohol, (**e**) D-galactosamine, (**f**) lipopolysaccharide, (**g**) thioacetamide, and (**h**) rifampicin.

In HBV-induced hepatitis, 2% (31) were common in both the literature review and the GeneCards database, while for acetaminophen, isoniazid, alcohol, D-galactosamine, lipopolysaccharide, thioacetamide, and rifampicin the common genes between the literature review and the GeneCards database were found to be 9.2% (14), 5.6% (4), 8.8% (33), 15.3% (11), 9% (25), 10.5% (6), and 5.6% (3), respectively. Figure 2 represents the common genes between the literature review and GeneCards.

**Figure 3:**
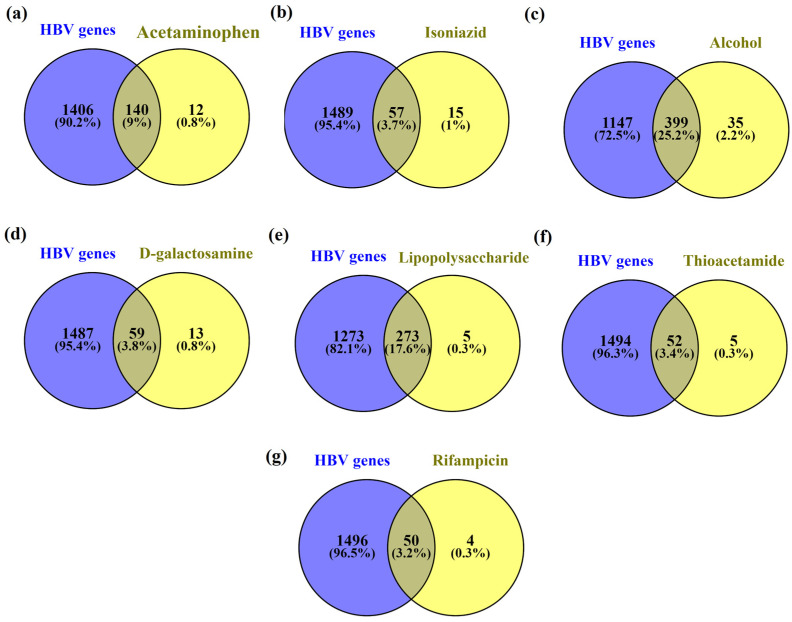
Common genes between HBV (**a**) acetaminophen, (**b**) isoniazid, (**c**) alcohol, (**d**) D-galactosamine, (**e**) lipopolysaccharide, (**f**) thioacetamide, and (**g**) rifampicin.

Further, the common genes present in HBV and acetaminophen, isoniazid, alcohol, D-galactosamine, lipopolysaccharide, thioacetamide, and rifampicin-induced hepatitis were found to be 9% (140), 3.7% (57), 25.2% (399), 3.8% (59), 17.6% (273), 3.4% (52) and 3.2% (50) respectively. Among them, HBV genes with alcohol genes had the highest similarity i.e., 25.2% whereas lipopolysaccharides had the second highest similarity i.e., 17.6%. Figure 3 represents the common genes between HBV and chemical-induced hepatitis.

### 2.3. Functional Enrichment Analysis to Assess the Hepatotoxicity

Initially, HBV, acetaminophen, isoniazid, alcohol, D-galactosamine, lipopolysaccharide, thioacetamide, and rifampicin were identified to modulate 1546, 152, 72, 434, 72, 278, 57, and 54 genes, respectively. The enrichment analysis of these individual sets of the gene revealed 217, 185, 184, 200, 185, 202, 167, and 172 molecular pathways, respectively. Supplementary Tables S9–S16 represent the molecular pathways modulated by HBV and chemicals.

**Figure 4:**
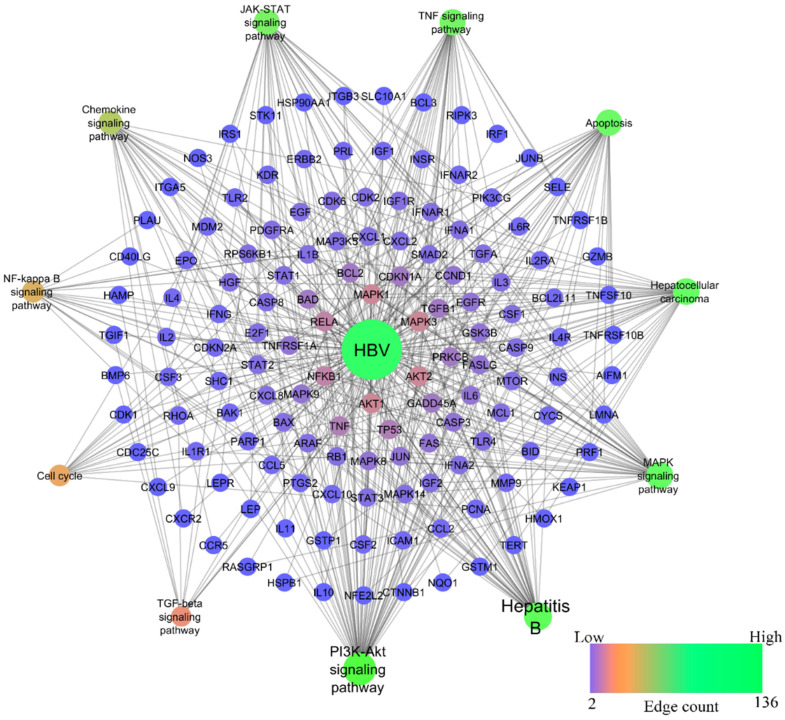
Network representation of HBV-modulated genes and pathways.

In the HBV-induced hepatitis model, out of 217 pathways modulated, nine pathways—namely, PI3K-Akt, TNF, JAK-STAT, MAPK, Chemokine, NF-kappa B, TGF-beta signaling pathways, Apoptosis, and Cell cycle—were prioritized to compare with chemically-induced hepatitis, as these pathways were significantly associated with the progression of hepatocellular carcinoma induced by HBV (refer KEGG ID: hsa05161). Among them, the PI3K-Akt signaling pathway scored the lowest FDR of 1.39E−33 and the highest gene count of 57, whereas TNF, JAK-STAT, MAPK, Chemokine, NF-kappa B, TGF-beta signaling pathways, Apoptosis, and Cell cycle scored the lowest FDR of 1.56E−27, 1.38E−22, 5.4E−20, 7.77E−13, 6.49E−12, 7.26E−27, 4.34E−09, respectively, and gene counts of 34, 33, 38, 24, 18, 11, 35, and 16, respectively. Figure 4 represents the network of HBV-modulated genes and pathways.

**Figure 5:**
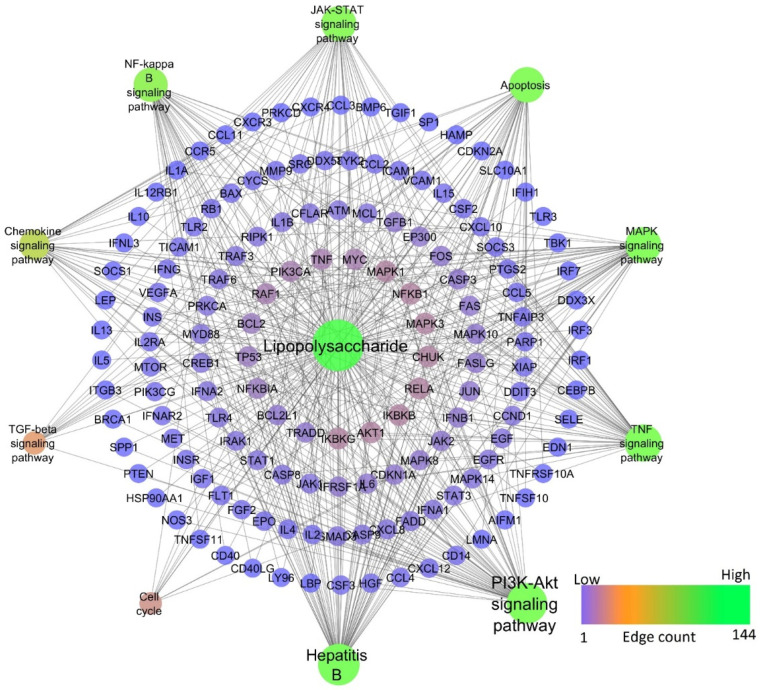
Network representation of lipopolysaccharide-modulated genes and pathways.

Among chemical-induced, lipopolysaccharide-induced hepatitis had the highest similarity compared to HBV-induced, whereas alcohol-induced hepatitis was found to be the second highest similarity with HBV. Table 1 represents the pathways modulated by HBV and selected chemicals. Lipopolysaccharide was found to modulate 202 molecular pathways, in which the PI3K-Akt signaling pathway scored the lowest FDR of 2.46E−41 and the highest gene count of 58, whereas TNF, JAK-STAT, MAPK, Chemokine, NF-kappa B, TGF-β signaling pathways, apoptosis, and cell cycle scored the lowest FDR of 4.49E−46, 1.48E−35, 5.8E−30, 3.71E−21, 4.11E−38, 0.000000036, 2.35E−38, and 7.55E−08 and gene counts of 45, 41, 44, 30, 38, 12, 41, and 13, respectively. Figure 5 represents the network of lipopolysaccharide-modulated genes and pathways.

**Figure 6:**
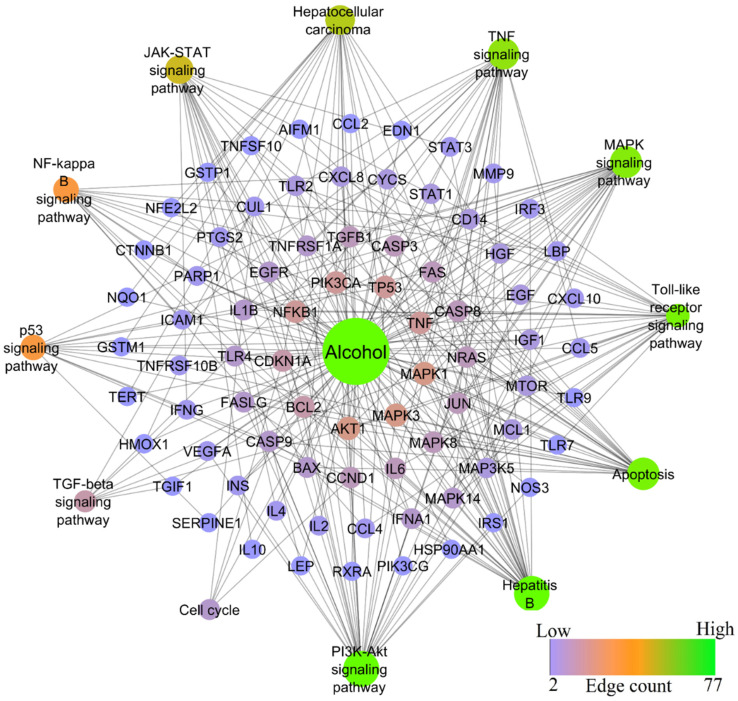
Network representation of alcohol-modulated genes and pathways.

Similar to HBV-induced hepatitis, alcohol-induced hepatitis shared the second-highest degree of similarity. Alcohol was found to modulate 200 molecular pathways, in which the PI3K-Akt signaling pathway scored the lowest FDR of 2.87E−21 and the highest gene count of 31, whereas TNF, JAK-STAT, MAPK, Chemokine, NF-kB, TGF-beta signaling pathways, Apoptosis, and Cell cycle scored the lowest FDR of 5.92E−23, 2.93E−14, 4.07E−16, 3.25E−10, 3.10E−11, 3.36E−05, 3.77E−24, 0.0063 and gene counts of 23, 18, 24, 15, 13, 7, 25, 5, respectively. Figure 6 represents the network of alcohol-modulated genes and pathways.

## 3. Discussion

Animal models are widely used to study the pathophysiology of chronic hepatitis B and to develop new drugs or treatment methods [234]. HBV can only infect humans and chimpanzees [235]. However, due to ethical and practical concerns, chimpanzees are not commonly utilized in HBV research [235]. Additionally, efforts have been conducted to spread HBV to smaller non-human primates. The tree shrew is the only rodent other than a primate that has been found to be susceptible to HBV infection, but the in vivo system still needs major improvement [7,236]. As a result of the absence of viral entry, cccDNA synthesis, and viral dissemination, mice with the HBV genome transfected, transduced, or transgenic can only support HBV replication, leaving the HBV life cycle unfinished. When human liver cells that maintain HBV infection are transplanted into immuno-deficient mice, the animals show apparent immunodeficiency, and their maintenance systems are very sophisticated [237]. As a result, the majority of gains in HBV research have been made utilizing mice models of HBV replication or infection, or models of HBV-related hepadnaviral infection [236,237,238].

**Figure 7:**
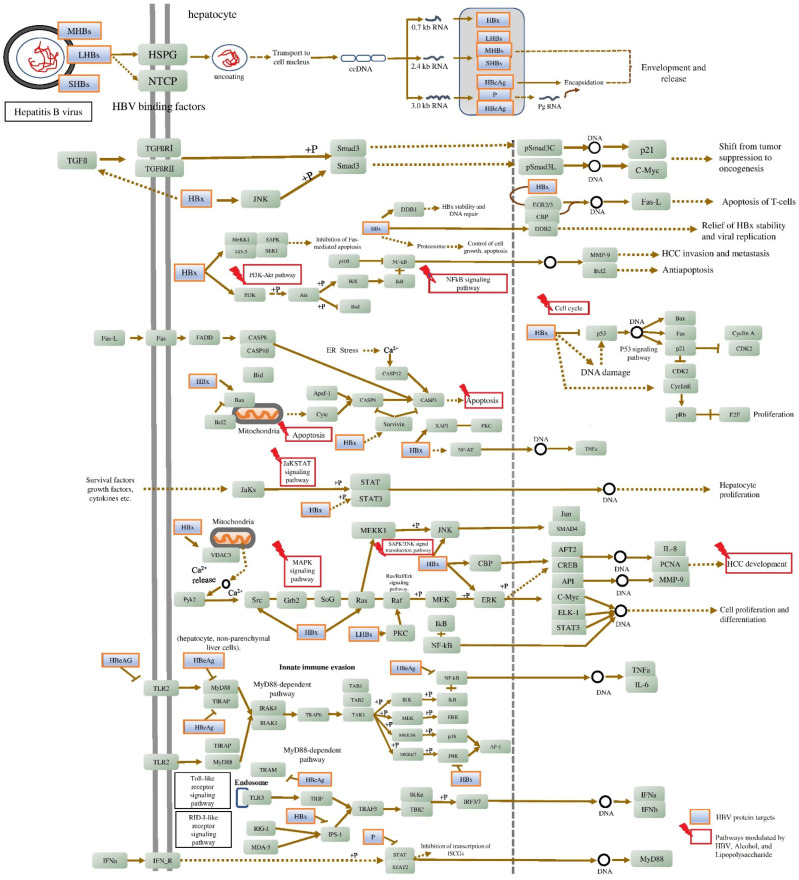
Molecular pathways triggered by alcohol and LPS resembled the HBV pathogenesis. The figure information is retrieved from the KEGG database “Hepatitis B: hsa05161”.

In line with previous investigations, some of the drugs used to cause hepatitis in rats are known to cause pathophysiology that is comparable to the pathogenesis of HBV in people [239,240,241]. Therefore, the goal of the current study was to use gene set enrichment and network pharmacology analysis to find a chemically induced hepatitis model that is similar to HBV pathogenesis. The study determined that the pathogenesis of HBV is similar in the alcohol- and LPS-induced hepatitis models. About 42 and 1538 genes were first gathered for HBV from the literature and GeneCards, respectively, of which 31 genes (2%) were common. In the enrichment analysis, 1546 genes were involved in 217 molecular pathways, in which nine pathways—namely, PI3K-Akt, TNF, JAK-STAT, MAPK, Chemokine, NF-kappa B, TGF-beta signaling pathways, Apoptosis, and Cell cycle—were majorly associated with HBV infection (KEGG ID: hsa05161). These pathways were significantly targeted by both LPS and alcohol (Figure 7 and Table 1).

The PI3K/Akt signaling pathway is associated with a variety of biological processes caused by enzymes, including glucose metabolism. Phosphatidylinositol 3-kinase (PI3K)/protein kinase B (AKT) and mitogen-activated protein kinase (MAPK) signaling are examples of signal transduction cascades that could be activated by HBV HBx, which is primarily present in the cytoplasm [242]. Alcohol inhibits the liver’s insulin signaling pathway, which leads to irregularities in the metabolism of glucose and lipids. This is one of the key factors contributing to the development of alcoholic liver disease (ALD) [243]. Alcohol was found to increase apoptotic expression and PI3K/Akt signaling while lowering hepatic perfusion, hence promoting cirrhosis [244]. Similarly, LPS is also reported to activate the PI3K/Akt and MAPKs [245]. In this study, HBV, alcohol, and LPS targeted the PI3K-Akt signaling pathway by modulating 57, 31, and 58 genes and the MAPK signaling pathway by 38, 24, and 44 genes, respectively. Among them, AKT1, AKT2, MAPK1, MAPK3, NFKB1, TNF, and BCL2 genes were identified as hub genes within the network. The activation of these signal pathways may contribute to liver cell malignant transformation.

TNF-α, one of the most important inflammatory cytokines, was first identified as an anti-tumor cytokine that resulted in tumor necrosis. Inflammation is fundamentally mediated by TNF-α, which also promotes the growth of cancers. Researchers have found that compared to healthy liver tissue, HCC expresses TNF-α at substantially higher levels [246]. TNF-α is a potent NF-kB signaling activator; during HBV infection, it increases HBx intracellular concentration by enhancing its stability and is essential for the onset and progression of HCC [247], whereas in alcohol-induced hepatitis, alcohol increases hepatocytes’ susceptibility to TNF-α-induced apoptosis. TNF-α levels were higher in both chronic drinkers and animal models fed alcohol over an extended period of time. In all kinds of liver cells, the NF-kB is a key regulator of cellular stress. In the cytoplasm of dormant cells, the family of NF-kB proteins, including RelA/p65, RelB, c-Rel, and p50, exist as dimers in a complex with inhibitory kB molecules [248]. Chronic alcohol use is thought to prime the liver by inducing basal and LPS-stimulated TNF-α and persistent NF-kB activation [249]. Hepatic macrophages’ expression of pro-inflammatory mediators is significantly regulated by NF-kB [249]. The activation of TLR4 by circulating LPS on liver macrophages, which results in NF-B activation and the generation of pro-inflammatory cytokines, is linked to chronic alcohol-mediated liver damage [250]. In this study, HBV, alcohol, and LPS targeted the TNF-α signaling pathway by modulating 34, 23, and 45 genes and the NF-kB signaling pathway by 18, 5, and 13 genes, respectively. The LPS has the lowest FDR score for TNF and NF-kB signaling pathways, i.e., 4.49E−46 and 4.11E−38, respectively. While for HBV and alcohol the FDR score for the TNF signaling pathway was 1.56E−27 and 5.92E−23, for the NF-kB signaling pathway it was 6.49E−12 and 3.10E−11, respectively. This indicates LPS possesses a significant effect on TNF and NF-kB signaling pathways. TGF-β a crucial cytokine that promotes fibrosis in a variety of chronic liver disorders and HCC. Overactivation of the TGF-β signaling pathway increases cell migration and invasion. The HBV HBx upregulates TGF-β on HCC progression by downregulating protein phosphatase magnesium-dependent 1A (PPM1a) [251]. Alcohol and LPS are also reported to increase the TGF-β and are prevalent in ALD. In this study, HBV, alcohol, and LPS targeted TGF-β signaling pathways by modulating 11, 7, and 12 genes, respectively.

Among the HBV proteins, HBx is the one that has been most commonly linked to the suppression of apoptosis and the stimulation of HCC development. Through the overexpression of PI3K and the stimulation of Akt phosphorylation, HBx stimulates the phosphatidylinositol-4,5-bisphosphate 3-kinase-protein kinase B (PI3K-Akt) pathway to suppress apoptosis. Drp-1 and Parkin are brought to the mitochondria by HBx to promote mitochondrial fission and mitophagy, which suppresses the intrinsic apoptotic pathway. Additionally, the activation of Akt inhibits BAD from moving to the mitochondria and apoptosis from occurring. HBx stimulates the nuclear factor kappa-light-chain-enhancer of activated B cells (NF-κB) signaling by degrading IκB. HBV may reduce the activity of the kinase that activates JNK in the MAPK-JNK pathway [252]. Similarly, chronic alcohol use reduces the mitochondrial maximum oxygen uptake rate, which in turn makes hepatocytes more vulnerable to alcohol-induced hypoxia and liver damage [253]. Similar to LPS, which is a highly pro-inflammatory molecule, endothelial responses to LPS include the production of cytokines, adhesion molecules, and tissue factors, as well as apoptotic endothelial cell death [254]. Activation of the NF-B TLR4/PI3K/Akt/GSK-3, cytokine, and other signaling pathways is how LPS most commonly causes apoptosis. The 35, 25, and 41 genes in the network were respectively targeted by HBV, alcohol, and LPS in this investigation to induce apoptosis.

The JAK/STAT signaling system is crucial for several physiological processes, such as cell division, stem cell maintenance, differentiation, and immune/inflammatory response control. Additionally, it has been shown that JAK/STAT signaling controls gluconeogenesis and liver regeneration. Different cytokines and growth factors, including interleukins, interferons, and members of the EGF family also activate the JAK/STAT pathway by binding to their respective transmembrane receptors. The current study reports that the HBV, alcohol, and LPS modulate 33, 18, and 41 genes to modulate the JAK/STAT pathway. It is well known that chronic alcohol use and LPS decrease ILs and IFN-induced STAT1 activation, which in turn lowers NK cell function in the liver and speeds up the development of hepatic fibrosis. STATs activation via ILs and IFN is necessary for hepatic regeneration [250]. However, investigation has revealed that HBV HBx also controls cellular growth and death in addition to having a significant impact on the innate immune response and viral replication. HBX controls the activity of JAK1, JAK2, and TYK2. Cho et al. indicated that HBX may prevent TYK2 activation, lowering the expression of the IFN- receptor 1 (IFNAR1) and preventing signal transduction mediated by exogenous IFNs [255]. The HBX-mediated interaction of SH2 domain-containing 5 (SH2D5) with transketolase (TKT) may activate STAT3 to increase HCC cell proliferation, and HBx was also reported to drive SH2D5 expression in HCC cells. IL-6 is essential for STAT3 activation. As it is, HBX has been shown to increase IL-6 expression in hepatoma cells [255]. On the other hand, alcohol and LPS are also well reported to increase the level of IL-6 and IL-6-facilitated acute inflammatory response in the liver, causing the development of chronic liver injury [256,257]. The researchers also identified that the liver damage in IL-6 knockout mice after alcohol feeding may be due to STAT3-independent signaling of IL-6 in hepatocytes. Hence, this confirms that IL-6 mediated liver damage is due to STAT3 activation [258]. On looking into the overall outcome of the study, HBV and chemicals cause hepatocellular carcinoma (HCC) through a multifactorial process and molecular pathways. Animal models of chemically induced HCC resemble hepatocarcinogenesis of HBV and this research sheds light on the screening of novel anti-HBV and hepatoprotective molecules using alcohol and LPS as a chemical-induced hepatitis model.

## 4. Materials and Methods

### 4.1. Identification of HBV-Associated and Chemically Induced Hepatitis Genes

A peer review of the literature and GeneCards database were used to collect the information on genes that are modulated by HBV and the selected chemicals to produce hepatitis, namely, acetaminophen, isoniazid, alcohol, D-galactosamine, lipopolysaccharide, thioacetamide, and rifampicin were selected to compare with the HBV-induced hepatitis. In the GeneCards database, the genes with a relevance score ≥20 were considered for evaluation to obtain the most relevant data. Here, we set a relevance score ≥20 cut-off to avoid the large number of genes that cause errors during enrichment analysis. Further, Venny 2.1 [259] was utilized to identify the common genes between the literature and GeneCards with HBV and chemically-induced hepatitis. In addition, the Kyoto Encyclopedia of Genes and Genomes (KEGG) database accession number “hepatitis B: hsa05161” was utilized to collect the molecular pathway regulated in HBV infection.

### 4.2. Gene set Molecular Pathway Enrichment Analysis

The set of genes collected for HBV and chemical-induced hepatitis were submitted to the STRING database [260]. The set of gene-regulated molecular pathways was retrieved from the STRING database inbuilt KEGG pathway database [261]. Further, the obtained list of pathways of HBV-induced hepatitis was matched with pathways collected from “KEGG ID: hsa05161” and finalized with the matched pathways for HBV-modulated pathways for further analysis. In a similar manner, the list of pathways for chemically induced hepatitis and the HBV pathways were compared for similarity based on gene counts and false discovery rate (FDR) [262,263].

### 4.3. Network Construction

The network between HBV and chemicals with their targets (involved in hepatitis) and the regulated pathways were constructed using Cytoscape (https://cytoscape.org/ (accessed on 20 February 2023)) version 3.6.1 [264]. The constructed network was recognized as directed and inspected by translating node size and color to low values corresponding to small sizes and bright colors toward the edge count. In addition, the edge size and color were mapped to edge betweenness, with low values corresponding to small sizes and low values equating to bright colors [265,266].

## 5. Conclusions

The GeneCards database was utilized in the current investigation to collect genes affected by HBV and several substances thought to induce hepatitis. It also underwent a thorough peer review process. Out of seven chemically induced hepatitis cases, alcohol- and LPS-induced hepatitis were found to share similar molecular pathways with HBV-induced hepatitis, according to gene set enrichment and network pharmacology analysis. Apoptosis, Cell cycle, PI3K-Akt, TNF, JAK-STAT, MAPK, Chemokine, NF-kappa B, and TGF-β signaling pathways were the major pathways modulated by HBV, which were also targeted by alcohol and LPS with significant gene counts and FDR scores, since alcohol is used to investigate chronic hepatitis and LPS is used to examine acute hepatitis. In contrast to HBV-induced hepatitis in rodents, alcohol-induced chronic hepatitis may be the option to study chronic hepatitis in rodents.

## Figures and Tables

**Table 1:** Functional enrichment analysis of genes modulated by HBV and selected chemicals.

Hepatitis Model		HBV		Acetaminophen		Isoniazid		Alcohol		D-galactosamine		Lipopolysaccharide		Thioacetamide		Rifampicin	
KEGG ID	Pathway Description	GC	FDR	GC	FDR	GC	FDR	GC	FDR	GC	FDR	GC	FDR	GC	FDR	GC	FDR
hsa05161	Hepatitis B	42	6.6E−32	21	3.46E−19	19	7.91E−22	30	8.48E−29	28	8.90E−36	66	1.14E−68	18	1.17E−21	18	3.57E−22
hsa04151	PI3K-Akt signaling pathway	57	1.39E−33	23	2.02E−15	14	1.02E−10	31	2.87E−21	20	3.35E−17	58	2.46E−41	16	5.34E−14	16	1.78E−14
hsa04668	TNF signaling pathway	34	1.56E−27	9	2.56E−07	7	5.89E−07	23	5.92E−23	14	2.21E−16	45	4.49E−46	9	3.49E−10	11	2.34E−13
hsa04210	Apoptosis	35	7.26E−27	10	8.51E−08	9	5.93E−09	25	3.77E−24	15	6.20E−17	41	2.35E−38	9	1.16E−09	13	1.89E−15
hsa05225	Hepatocellular carcinoma	36	1.35E−25	16	2.12E−13	14	8.53E−15	20	1.56E−16	18	4.48E−20	30	8.42E−23	12	6.46E−13	12	2.65E−13
hsa04630	JAK-STAT signaling pathway	33	1.38E−22	12	4.22E−09	8	3.73E−07	18	2.93E−14	12	8.40E−12	41	1.48E−35	12	6.46E−13	9	3.01E−09
hsa04010	MAPK signaling pathway	38	5.4E−20	16	4.47E−10	12	1.80E−09	24	4.07E−16	19	2.32E−17	44	5.8E−30	12	2.56E−10	16	1.74E−15
hsa04062	Chemokine signaling pathway	24	7.77E−13	8	7.08E−05	4	0.0072	15	3.3E−10	11	7.06E−10	30	3.71E−21	8	2.68E−07	6	3.44E−05
hsa04064	NF-kappa B signaling pathway	18	6.49E−12	7	1.39E−05	4	0.00094	13	3.10E−11	8	2.50E−08	38	4.11E−38	3	0.0075	6	1.54E−06
hsa04110	Cell cycle	16	4.34E−09	5	0.0023			5	0.0063	6	2.06E−05	13	7.55E−08	3	0.0115	5	5.74E−05
hsa04350	TGF-beta signaling pathway	11	0.00000324	ND		3	0.0075	7	3.36E−05	ND		12	0.000000036	3	0.00041	4	0.0045

ND, Not Detected; GC, Gene Count, reprents the number of set of genes within the pathway of individual model; FDR, False Discovery Rate, discribes the significance of each pathway.

## Data Availability

The authors confirm that the data supporting the findings of this study are available within the article (and/or) its Supplementary Materials.

## Supplementary Materials

The following supporting information can be downloaded at: https://www.mdpi.com/article/10.3390/ijms241311146/s1.
